# Affording Childcare on a Surgical Resident’s Salary

**DOI:** 10.1001/jamanetworkopen.2025.0708

**Published:** 2025-03-13

**Authors:** Margaret G. Mercante, Emily G. Tocco, Nidhi Kuchimanchi, Mohamad El Moheb, Maria F. Nunez, Mackenzie M. Mayhew, Susan J. Kim, Allan Tsung, Lily S. Cheng, Russell G. Witt

**Affiliations:** 1 Department of Surgery, University of Virginia, Charlottesville

## Abstract

**Importance:**

Previously published literature found that 28.6% of surgical residents have or are expecting children, yet little information exists regarding the financial demands of childcare during residency.

**Objective:**

To evaluate surgical residents’ net financial balance after childcare costs at various postgraduate years and child ages.

**Design, Setting, and Participants:**

This cross-sectional study, conducted from June 14 to August 2, 2024, examined surgical residency programs across the US using publicly available data. Programs were categorized into US regions based on the Association of American Medical Colleges classifications: Northeast, Midwest, South, and West. Childcare costs were obtained from the National Database of Childcare Prices, and annual expenditure data came from the Bureau of Labor Statistics.

**Main Outcomes and Measures:**

The primary outcome was residents’ net income by year of residency, calculated using salaries and expenditures. To compare costs by region and child age, net income was determined by subtracting mean expenditures and childcare costs from residency salaries. Calculations were validated using the Massachusetts Institute of Technology Living Wage Calculator.

**Results:**

Of 351 US surgical residency programs, 295 with publicly available salaries for postgraduate years 1 through 5 met inclusion criteria. A total of 290 programs (98.3%) showed a negative net income when expenditures and childcare costs were deducted. This finding held true across all child age groups and US regions. The West had the most negative mean net income (−$18 852 [range, –$35 726 to $766]), followed by the Northeast (–$15 878 [range, –$37 310 to $3589]), Midwest (–$12 067 [range, –$26 111 to $1614]), and South (–$8636 [range, –$18 740 to $4826]). Parents of school-aged children in the South had the lowest mean negative net income (−$8453 [range, −$16 377 to $3417]), while parents of infants in the West had the highest mean negative net income (−$21 278 [range, −$35 726 to −$5112]).

**Conclusions and Relevance:**

This cross-sectional study of surgical residents’ net income found that, after accounting for mean annual expenditures and childcare costs, a surgical resident’s salary was insufficient to cover living expenses and childcare costs for single resident parents. This financial obstacle may deter individuals from pursuing surgical residency or from starting families as surgical residents.

## Key Points

**Question:**

Is it financially feasible to support a child solely on a surgical resident’s salary?

**Findings:**

This cross-sectional study including 295 surgical residency programs in the US found that 98% of programs left residents with a net negative income after accounting for annual expenses and infant childcare costs.

**Meaning:**

The findings of this study suggest that financially supporting a child solely on a resident’s salary is not feasible and that outside support is necessary for surgical resident parents.

## Teaser

This cross-sectional study evaluates surgical residents’ net financial balance after childcare costs at various postgraduate years and child ages.

## Introduction

Parenthood poses a significant challenge for physicians during residency. This is especially true for surgical residencies, which require 5 or more years of training that coincide with the typical age range for starting families. A survey conducted between 2010 and 2017 of 2 tertiary care centers found the mean age of surgical residents to be 30.4 years, ranging from 25 to more than 33 years.^1^ Moreover, the Centers for Disease Control and Prevention reported in 2021 that the mean age of mothers at first birth in the US was 27.3 years.^2^ Literature suggests that the difficulty of balancing parenthood with surgical training often deters women from pursuing surgical residency.^3^ Pregnancy during residency is associated with increased risk of infertility, pregnancy complications, and decreased job satisfaction,^4,5,6^ demonstrating a negative physiological and emotional effect of surgical training on childbearing. Li et al^7^ found that parenthood-based mistreatment, particularly among surgical residents, is likely associated with gendered attrition in the field. This work-life imbalance also applies to the nonchildbearing parent; a recent study reported the median duration of nonchildbearing paternity leave was 7 days, leaving over half of participants dissatisfied with their leave duration.^8^ Men may feel deterred from taking longer paternity leave, fearing negative reactions from program directors and colleagues.^9^ However, there are currently limited data on the economic hardships of parenting during surgical training.

Childcare costs become significantly more burdensome for residents, who often work twice the typical 40-hour US workweek while earning incommensurate salaries.^10,11^ According to the Association of American Medical Colleges (AAMC) 2022 resident survey, the mean resident salary was $58 921, with residency salaries increasing only marginally over the last decade and failing to keep pace with cost-of-living increases.^12^ Although the medical community is well aware of the disparity between resident salaries and their working hours, few studies have explored how these salaries compare with childcare expenses and living wages. This study sought to offer a relative estimate of the financial burden of supporting a family during surgical residency.

## Methods

This cross-sectional study was conducted from June 14 to August 2, 2024, with publicly available data on surgical residency programs, childcare costs based on zip code, and mean annual expenditures for geographical regions in the US. We defined the different regions based on AAMC classifications of Northeast, Midwest, South, and West.^13^ This study received an exemption from the University of Virginia institutional review board because all data were publicly available. It was conducted in accordance with the Strengthening the Reporting of Observational Studies in Epidemiology (STROBE) reporting guideline for cross-sectional studies.

We compiled a list of surgical residency programs in the US using Doximity,^14^ a residency navigator, and subsequently reviewed the individual programs’ websites to obtain recent salary amounts for each postgraduate year. Programs were included in our study if their residency salaries were publicly available and excluded if salaries were unavailable to the public or if they did not have childcare costs for their respective counties included in the National Database of Childcare Prices (NDCP).^15^ The main hospital sites of each program were used to determine the county, state, and zip code for calculation of expenses. Salaries were averaged across postgraduate year 1 (PGY1) through PGY5 for each program to use for net income calculations. Childcare costs for each childcare age group, including infants (birth to 23 months), toddlers (24-35 months), preschool-aged children (36-54 months), and school-aged children (≥55 months), were obtained for each county from the NDCP based on 2023 childcare price data.^15^ The database included estimated childcare prices for both home-based and center-based care for each age group. These prices were averaged to provide the childcare costs used in our final calculations.

The most recent mean annual expenditures for 2022 were retrieved from the Bureau of Labor Statistics (BLS) for each US region, including a breakdown of spending on transportation, personal taxes, housing, food, and other categories.^16^ Our “other” category includes all categories included in the US BLS annual expenditure data not specifically listed in our breakdown of expenditures.

To validate our model and provide an additional estimate of expenditures, the Living Wage Calculator developed by the Massachusetts Institute of Technology (MIT) was also used to analyze the living wage of each residency program’s county and residual income after accounting for childcare costs from the NDCP.^17^ We included the following factors from the MIT Living Wage Calculator: annual expenditures (total), transportation, taxes, housing, food, other, and childcare costs. Similar to our methods, the MIT Living Wage Calculator used the US BLS Consumer Expenditure Survey to define their “other” category.

To compare costs between regions and between child age groups, we calculated the net income by deducting mean annual expenditures and childcare costs from the mean resident salary for each program. Findings for each child age category were averaged across the 4 different US regions: Northeast, West, Midwest, and South. The residual income after accounting for childcare expenses was calculated for each program by subtracting the mean childcare costs by zip code from the mean surgical resident’s salary at that program.

### Statistical Analysis

Descriptive statistics were used to summarize residency program salaries, childcare costs, and annual expenditures across the included regions. Mean values and ranges were calculated for salaries (PGY1-PGY5), childcare costs (infants, toddlers, preschool-aged children, and school-aged children), and annual expenditures using data from the NDCP, BLS, and MIT Living Wage Calculator. Net income was calculated by subtracting annual expenditures and childcare costs from residency program salaries. Regional differences in net income were assessed by averaging values across the 4 US regions (Northeast, Midwest, South, and West), as defined by the AAMC.

To validate the robustness of our financial estimates, we conducted a comparative analysis using both BLS expenditure data and the MIT Living Wage Calculator. A sensitivity analysis was performed to evaluate differences in net income when considering home-based vs center-based childcare costs. Data were processed and analyzed using R Studio, version 2024.12.0+467 (R Project for Statistical Computing) and Prism, version 10.0.0 (GraphPad Software) for descriptive statistics. No inferential statistical tests were conducted, as the study focused on a descriptive evaluation of financial burdens rather than hypothesis testing.

## Results

Of the 351 surgical residency programs in the US, 295 programs with publicly available PGY1 through PGY5 residency salaries that matched our inclusion criteria were included in our study. We excluded the 56 programs that either did not disclose their residency salaries online or did not have childcare costs for their respective counties included in the NDCP. Mean salaries across PGY1 and PGY5 years were calculated for net income calculations (Table).

**Table.** Costs of Transportation, Taxes, Housing, Education, Food, Other Expenditures, and Annual Childcare Expenses for Children Across Residency Programs^a^

Characteristic	Northeast	Midwest	South	West
Salary, mean (range), $				
PGY1	70 085 (56 797 to 84 315)	63 386 (48 144 to 74 624)	60 674 (52 624 to 69 774)	70 711 (56 834 to 89 261)
PGY5	83 179 (65 480 to 103 800)	72 748 (51 714 to 87 376)	70 404 (59 000 to 82 717)	83 841 (65 549 to 101 434)
Mean PGY1-PGY5	76 732 (59 348 to 95 467)	67 888 (49 521 to 80 837)	65 225 (57 000 to 75 845)	76 576 (60 984 to 95 051)
Annual expenditure, $				
BLS	79 741	67 870	65 576	83 317
MIT Living Wage Calculator, mean (range)	80 758 (59 764 to 102 791)	67 246 (60 353 to 78 700)	66 078 (57 270 to 78 008)	83 124 (67 527 to 101 057)
Transportation, $				
BLS	12 093	11 912	11 932	13 420
MIT Living Wage Calculator, mean (range)	9910 (4560 to 13 013)	10 791 (9315 to 12 678)	10 995 (9313 to 14 356)	12 029 (8071 to 17 289)
Taxes, $				
BLS	14 898	10 257	7976	13 064
MIT Living Wage Calculator, mean (range)	14 087 (8189 to 21 586)	10 066 (6470 to 13 823)	8687 (5813 to 17 262)	13 316 (8313 to 19 686)
Housing, $				
BLS	27 433	21 907	21 494	28 938
MIT Living Wage Calculator, mean (range)	23 716 (10 192 to 38 369)	15 042 (10 380 to 20 151)	17 468 (8430 to 26 850)	26 040 (14 360 to 37 778)
Food, $				
BLS	10 199	8827	8443	10 699
MIT Living Wage Calculator, mean (range)	6995 (5729 to 9898)	6389 (5630 to 7523)	6346 (5115 to 10 917)	6557 (5829 to 8536)
Other, $				
BLS	12 952	15 484	14 838	15 870
MIT Living Wage Calculator	7884	7294	6966	8459
Annual childcare cost, mean (range), $				
Infant	14 792 (8236 to 23 136)	11 628 (5762 to 17 291)	9694 (5145 to 17 396)	14 538 (9947 to 23 888)
Toddler	13 388 (7032 to 21 392)	10 712 (5601 to 15 302)	8655 (5008 to 14 548)	12 172 (8842 to 20 248)
Preschool aged	12 238 (7032 to 18 056)	10 743 (5601 to 15 302)	8102 (4677 to 11 599)	11 798 (8263 to 20 248)
School aged	10 743 (4264 to 17 781)	8103 (4334 to 13 007)	6689 (3795 to 9793)	9936 (6710 to 15 765)
Net income, $				
National Database of Childcare Prices, mean (range)				
Infant	−17 802 (−37 310 to −2401)	−13 610 (−26 111 to −2015)	−10 045 (−18 740 to 1137)	−21 278 (−35 726 to −5112)
Toddler	−16 710 (−36 378 to 446)	−12 693 (−25 950 to −1870)	−9006 (−17 392 to 2209)	−18 913 (−35 726 to −1615)
Preschool aged	−15 247 (−35 235 to 1553)	−11 870 (−25 950 to −605)	−8453 (−16 377 to 3417)	−18 539 (−35 170 to −1615)
School aged	−13 753 (−35 147 to 3589)	−10 093 (−25 790 to 1614)	−7040 (−15 890 to 4826)	−16 677 (−33 567 to 766)
MIT Living Wage Calculator, mean (range)				
Infant	−18 830 (−51 723 to 5201)	−11 176 (−29 679 to 597)	−10 547 (−29 136 to 6898)	−19 993 (−42 243 to −5753)
Toddler	−17 426 (−50 791 to 5479)	−10 259 (−29 518 to 758)	−9508 (−27 788 to 7970)	−17 627 (−40 181 to −2652)
Preschool aged	−16 275 (−49 648 to 5642)	−9436 (−29 518 to 850)	−8955 (−26 634 to 7970)	−17 253 (−38 513 to −2652)
School aged	−14 781 (−49 560 to 6015)	−7659 (−29 358 to 3648)	−7543 (−25 017 to 7741)	−15 391 (−32 114 to −1961)

Abbreviations: BLS, Bureau of Labor and Statistics; MIT, Massachusetts Institute of Technology; PGY, postgraduate year.

^a^ Age ranges for children: infants, birth to 23 months; toddlers, 24-35 months; preschool-aged children, 36-54 months; and school-aged children, 55 months or older.

Using the methods described, a comprehensive table was created to illustrate the mean salaries, annual expenditures, and their breakdowns based both on BLS data and on MIT Living Wage Calculator cost data, mean childcare costs by child age group, and total net incomes for each child age group by region (Table). Based on these findings, a single-income surgical resident was unable to earn a living wage and care for an infant child during their surgical residency, with 290 of 295 programs (98.3%) leaving residents with negative net incomes (Table; Figure). Regional differences in net income (Figure) highlight that potential debt was greatest in the West (−$18 852 [range, –$35 726 to $766]), followed by the Northeast (–$15 878 [range, –$37 310 to $3589]), Midwest (–$12 067 [range, –$26 111 to $1614]), and South (–$8636 [range, –$18 740 to $4826]). An analysis of income by region using our model revealed that the West was the most expensive region for surgical residencies and the South the least. Parents in the South had the lowest mean negative net income for surgical residents, which was still −$8636 (range, −$18 740 to $4826) after accounting for childcare costs and living expenses, while parents of infants in the West had the highest mean negative net income (−$21 278 [range, −$35 726 to −$5112]) (Table). Across all regions, infant care was the most expensive type of childcare, followed by care for toddlers, preschool-aged children, and school-aged children.

**Figure.**
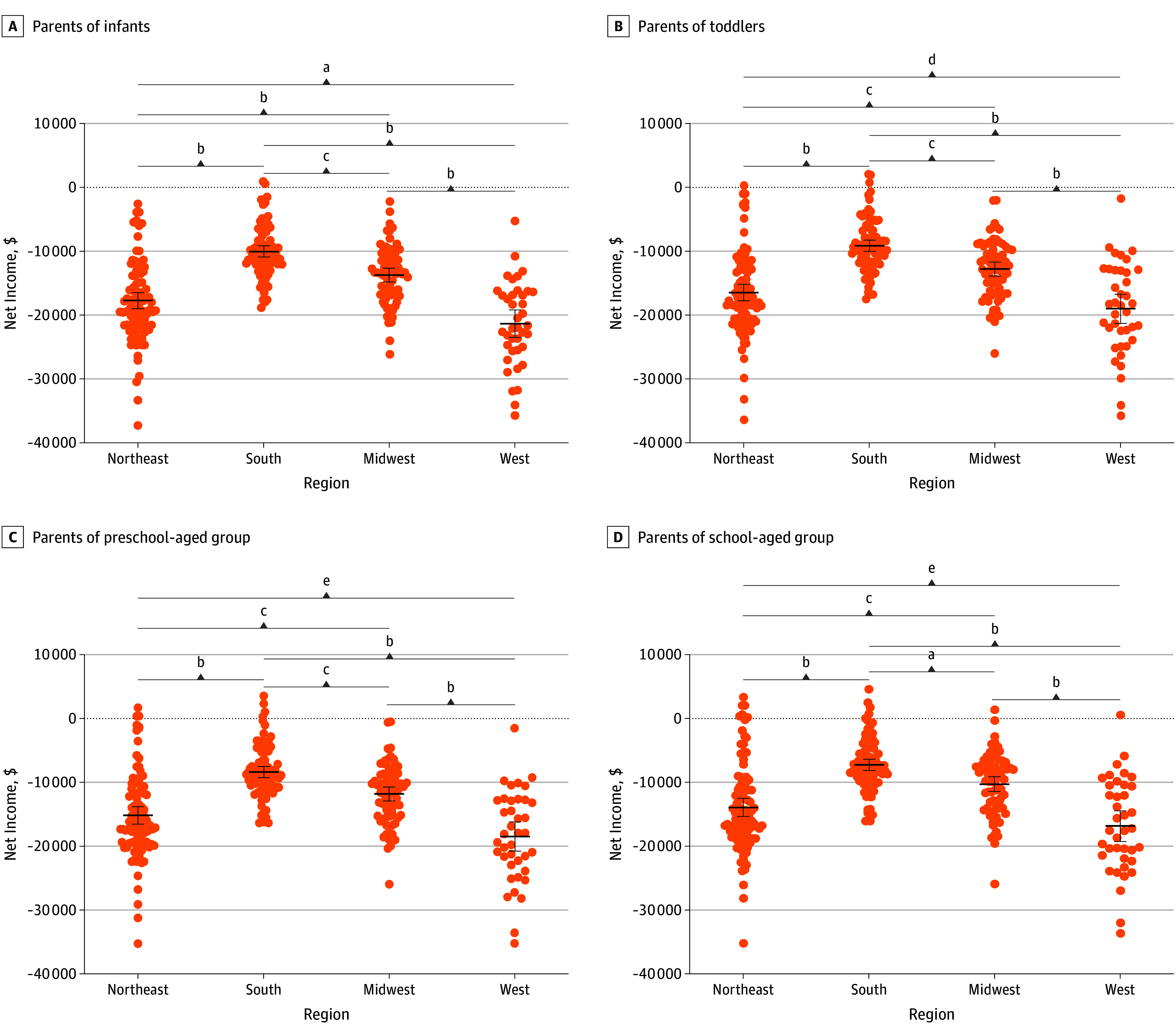
Net Income for Parents With Children Categorized as Infants (Birth to 23 Months), Toddlers (24-35 Months), Preschool Aged (36-54 Months), and School Aged (≥55 Months) Across General Surgery Residency Programs in the US ^a^*P* ≤ .01. ^b^*P* < .001. ^c^*P* ≤ .001. ^d^*P* = .08. ^e^*P* ≤ .05.

## Discussion

This study evaluates the affordability of having a child while earning a surgical resident’s salary. Our financial analysis revealed that 98.3% of residency programs left residents caring for children with a negative net income. These findings were consistent across geographical regions, with parents in surgical residencies in the West having the highest negative income. After deducting the cost of caring for an infant, most surgical resident salaries were too low to meet the minimum standard of living in their community, defined as the local wage rate that a full-time worker requires to cover the costs of their family’s basic needs where they live.^17^ To cover living expenses, a resident would need another source of income or to incur significant debt during the course of their training.

Although several studies have previously highlighted the difficulties of starting a family during surgical residency,^3,4,5,6,7,8,9^ few have addressed the economic challenges of parenthood. One recent study from Liles et al^18^ compared neurosurgical resident salaries at 118 programs with a calculated cost of living and similarly concluded that mean salaries precluded neurosurgical residents with children from earning a living wage. Our study used both the MIT Living Wage Calculator and NLBS data and took into account the large variations between surgical resident salaries and cost-of-living indices across the US^19^ to calculate the financial burden of childcare at 295 general surgery programs. Furthermore, our study considered childcare costs based on zip code for different age groups and includes salaries from each general surgery residency program. We concluded that cost of living is also prohibitively expensive for general surgical residents with children. In our study, we chose to examine surgical residents, who reported a mean of 84.3 weekly work hours, compared with 69.2 hours reported by internal medicine and neurology residents and 52.2 hours reported by anesthesiology and radiology residents.^20^ Thus, surgical residents work a mean of over 4 hours more than the weekly 80-hour work limit established by the Accreditation Council for Graduate Medical Education.^21^ Given the extended work hours, over double a typical 40-hour work week in nonmedical professions, we focused primarily on the burden of parenthood for surgical residents. However, as residents in other medical specialties also report work hours notably above the standard 40-hour work week, this topic is an important issue for most medical specialty trainees, and these findings likely hold true for those groups as well. To add to this financial burden, inflation increased on average 2.5% annually between 1990 and 2022,^19^ meaning that the cost of living increased 2.2 times faster than resident salaries over those 32 years. Only 33% of institutions accounted for cost of living when determining residents’ salary.^22^

Our calculations are based on a single child being supported by a sole income, which may not represent typical family dynamics. Spousal or family support, either financial or in the form of childcare, was not taken into account in our calculations and would likely introduce significant variability. However, as an approximation, according to the BLS, the median weekly earning of a full-time worker aged 25 to 34 years in 2024 was $1043, equating to an annual median wage of $57 356.^16^ Combining this income with a surgical resident’s salary and considering mean expenses and childcare costs, a typical 2-income family could avoid major debt but would still be left with very little remaining income to support one child, let alone multiple children.

The financial challenges of parenting during residency disproportionately impact trainees from socioeconomically disadvantaged backgrounds and underrepresented groups, deepening inequities within graduate medical education. Trainees without access to a working partner, family support, or subsidized childcare face unique barriers, as these resources often offset the high costs of childcare. For example, same-sex couples frequently incur additional expenses related to assisted reproductive technology or adoption, further compounding the financial strain. These disparities highlight systemic inequities in the ability to balance family responsibilities with the demands of residency, ultimately disadvantaging individuals from less-privileged backgrounds and potentially deterring them from pursuing surgical training

Our calculations also do not account for additional non–childcare-related financial burdens that surgical residents may face, such as loan repayment. In 2022, the mean education debt for medical students at the time of medical school graduation was reported to be about $200 000.^23^ Student loans can typically only be deferred for a maximum of 3 years, which is well below the duration of surgical training programs. Moreover, 80.4% of surgical residents chose to pursue additional years of training beyond residency,^24^ thereby extending the time that their income remains at trainee levels and loan interest accrues. Thus, the cost of child rearing during residency and fellowship training has the potential to add significant additional debt to an existing burden. Again, these issues may disproportionately affect trainees from socioeconomically disadvantaged backgrounds who may enter training with greater educational debt.

### Limitations

Our study has several important limitations, including the lack of available financial information across residency programs and national databases. Several residency programs were excluded from the analysis due to unavailable or incomplete salary information. In addition, the BLS does not publish information on annual living expenses by county, resulting in the use of county-level living expense data for our calculations. The annual expenditure categories used in our analysis reflect general population means and may not fully account for unique circumstances among surgical residents. Certain residency programs provide housing stipends, meal allowances, or transportation subsidies, which could partially offset the costs of living. A study on housing affordability among US medical residents found that housing stipends were available at 117 programs (13.7%).^25^

Overall, our study’s limitations likely result in an underestimate of the financial burden of childcare during residency and further underscore the need to better quantify and address this significant challenge. We obtained childcare costs from the NDCP that are based on a 40-hour work week, although residents have significantly longer hours and relative inflexibility in scheduling.^21^ Surgical residents’ extended work hours and irregular schedules often necessitate off-hours childcare, such as overnight or weekend care, which is typically more expensive and less available than standard childcare options. Although this study models absolute childcare costs based on mean expenses, it does not directly account for these unique challenges, which may further amplify the financial strain faced by resident parents.

Addressing the financial burden of childcare for surgical residents is pertinent for resident and family wellness, as childcare has been cited as one of the greatest challenges distracting residents from education and training.^26^ Several potential solutions have been previously proposed or implemented, including on-site childcare and stipends. Various hospital systems and businesses have implemented on-site daycare and subsidy programs to support workers with childcare costs.^4^ Furthermore, residency programs in other specialties are beginning to address financial burdens by offering childcare stipends that align with mean daycare expenses.^27^ Although these solutions may help alleviate the childcare burden for residents, they have significant limitations. For example, programs offering stipends typically provide only partial financial relief, and on-site childcare, while promising, faces practical challenges such as limited availability and issues of equity among the entire employee base. These constraints are particularly pronounced in larger, urban health care systems with higher childcare costs and greater demand. Unionized residency programs have shown some advantages in terms of nonmonetary benefits such as increased vacation time, housing stipends, and employer-sponsored health insurance; however, significant differences in salary between unionized and nonunionized programs have not been seen.^28,29^

Recognizing these many challenges, our aim is not to propose definitive solutions but to highlight the magnitude of the financial strain and its implications for trainees. Future efforts should focus on scalable strategies that address the unique needs of residents, such as childcare partnerships, expanded institutional support, and policy-driven subsidies that can alleviate these systemic pressures.

## Conclusions

In this cross-sectional study of surgical residents’ net income, when accounting for mean annual expenditures and childcare costs, surgical residents encountered a significant financial burden when starting families. Although the costs of living and childcare have increased, residency salaries have not kept pace, leaving parents struggling to afford childcare alongside other essential living expenses. In totality, surgical residents across the country frequently struggle to cover annual expenses on their salary alone, making the added burden of childcare costs unmanageable and potentially deterring individuals from pursuing surgical residencies or starting families during training. Residency programs and graduate medical education must address the financial hardship of child rearing when determining fair compensation for trainees.

## Notes

Supplement.
Data Sharing Statement
