# Supplementary material for: Affording Childcare on a Surgical Resident’s Salary
Source: JAMA Netw Open. 2025 Mar 13;8(3):e250708. doi: 10.1001/jamanetworkopen.2025.0708 (PMC11907318; doi:10.1001/jamanetworkopen.2025.0708)
Supplement: Supplement. — Data Sharing Statement [file jamanetwopen-e250708-s001.pdf]

Data Sharing Statement

Mercante. Affording Childcare on a Surgical Resident's Salary. *JAMA Netw Open*. Published March 13, 2025. doi:10.1001/jamanetworkopen.2025.0708

Data
Data available: No

Additional Information
Explanation for why data not available: Data is already publicly available
